# Renal manifestations and associated factors among HIV infected children at Muhimbili National Hospital, Dar es Salaam, Tanzania

**DOI:** 10.1186/1471-2334-12-S1-O11

**Published:** 2012-05-04

**Authors:** Francis Fredrick, Paschal Ruggajo, Eden E Maro, Bjarne Magnus Iversen, Gopel Basu

**Affiliations:** 1 Department of Pediatrics and Child Health, School of Medicine, Muhimbili University of Health and Allied Sciences, Dar es Salaam, Tanzania; 2 Department of Internal Medicine, School of Medicine, Muhimbili University of Health and Allied Sciences, Dar es Salaam, Tanzania; 3 Section of Nephrology, Haukeland University Hospital, Bergen, Norway; 4 Department of Nephrology, Christian Medical College Vellore, Tamil Nadu, India

## Background

Human Immunodeficiency Virus infection is a global challenge and sub-Saharan African countries contribute significantly to this pandemic. Children are vulnerable and acquire the infection mostly from their mothers. Highly Active Anti-retroviral Therapy have led to dramatic changes in the incidence of opportunistic infections with reduction in morbidity and mortality, which has paved way for manifestation of non-infectious complications including renal complications. The aim of this study was to determine prevalence of microalbuminuria, proteinuria and associated factors among Tanzanian children.

## Methods

We recruited 240 HIV infected children attending care and treatment clinic. Microalbuminuria and proteinuria were determined by using dipstick and Microalbumin 2-1 combo test strips on spot urine respectively. Serum Creatinine, white blood cell and CD4 counts were determined. Renal ultrasound examinations were also performed.

## Results

Forty nine children (20.4%) had microalbuminuria and 17 (7.1%) had proteinuria. Prevalence of proteinuria was significantly higher among children aged 120 months and above (p-value<0.05). Lower CD4 percent (< 25%) was a risk factor for microalbuminuria (p-value<0.01) and proteinuria (p<0.01). Mean CD4 count was significantly lower in children with microalbuminuria (p-value<0.05) and proteinuria (p-value <0.001). Twenty eight (11.7%) children out of 153 had increased cortical echogenicity on ultrasound examination.

## Conclusion

Proteinuria and increased cortical echogenicity were prevalent among HIV infected children who may indicate early onset of renal complications and this call for routine screening for early detection.

